# On Gonorrhœa in Its Constitutional Aspects

**Published:** 1889-06

**Authors:** 


### ON GONORRHŒA IN ITS CONSTITUTIONAL ASPECTS.

In reading the proof of the article having the above heading, by Dr. Burnett, in the present number of THE HOMŒOPATHIC PHYSICIAN, we were reminded of two or three interesting cases of gonorrhœa that have fallen under our own observation at different times.

One of these we recollect had a decidedly syphilitic aspect. There were chancroids along the tract of the urethra. There was contraction of the walls of the urethra, with consequent rupture and bleeding when spells of chordee occurred. A bubo started in the right groin, while a red line led to it from the end of the prepuce—which was rather long, and in a state of phimosis. The urging to urinate was very sudden and violent, and almost involuntary, with profuse flow as if from a force-pump.

All these symptoms seemed to call for Mercurius. We accordingly gave Mercurius-vivus$^{cm}$ (Fincke), and the violent symptoms subsided in twenty-four hours. We were not so fortunate in controlling the flow of mucus from the urethra, which continued some weeks longer. It was finally cured by Sulphur$^{2c}$ (B. & T.) in water.

Then followed a rheumatism which, rather singularly, was confined to the heels and mostly in the Tendo Achilles as far as we could discover. This was cured by Pulsatilla$^{2c}$ in three or four days. No condylomata followed during six months of our subsequent knowledge of the patient. After that period we lost sight of him.

Another case that occurs to us was in a young man who had had repeated attacks of gonorrhœa. When he contracted his latest attack he decided that Homœopathy was too slow for him.

So, notwithstanding our warnings, he consulted a physician of the old school of medicine. Injections were administered, and the usual result followed—stricture and condylomata. His physician treated the stricture with bougies. He proposed to "snip of" with the scissors the fig-warts, which formed a complete Elizabethan ruff around the head of the penis, when the patient happened to mention that his homœopathic doctor could cure such things with medicine. "Can he?" exclaimed the doctor in surprise; "I would like to see that done. Go see him and ask him to do it." The young man obeyed. He came to our office, told the facts, and desired treatment. He assured us that the bougie treatment had practically ended. So we gave him Sepia[2c] (Jenichen), and in two or three weeks they were all gone. The *regular* physician then sent the patient to our office again with a message inquiring what remedy it was that we gave, as he had a dozen men whom he "would put upon it tomorrow!!"

Yet another case came to hand. A young man who had had gonorrhœa treated with injections, consulted us for "piles." We took his word for it and gave several remedies that had no effect. We then did what we should have done at first. We made an examination and discovered the anus encircled with a ring of condylomata. The young man said he had "caught the piles from a water-closet!" After this discovery we gave Sepia, and in three weeks the fig-warts disappeared.

Our own supposition from the limited number of cases we have seen, some of which are not sufficiently interesting to relate, is that the fig-warts generally result from suppressing the urethral flow with astringent injections.

